# NSAIDs trigger drug-specific response to exercise: a multi-omics systems biology analysis of a randomized crossover trial

**DOI:** 10.1186/s13395-026-00435-4

**Published:** 2026-07-03

**Authors:** Brandon M. Roberts, Alexander B. Lawrence, Swapna Kannan, Alyssa V. Geddis, Cara E. Sczuroski, Jess A. Gwin, Candace Moyler, Allison Hoke, Aarti Gautam, Jeffery S. Staab, Ronald W. Matheny, Rasha Hammamieh, Nabarun Chakraborty

**Affiliations:** 1 US Army Research Institute of Environmental Medicine, Natick, MA USA; 2 Experimental Therapeutics Branch, CIDR, Walter Reed Army Institute of Research, Silver Spring, MD USA; 3 Medical Readiness Systems Biology, CMPN, Walter Reed Army Institute of Research, Silver Spring, MD USA; 4 Military Operational Medicine Research Program, Fort Detrick, MD USA; 5 Army Educational Outreach Program, 503 Robert Grant Ave, Silver Spring, MD 20910 USA

**Keywords:** Inflammation, Signaling, Musculoskeletal, Exercise, Myokines, Metabolomics

## Abstract

**Background:**

Non-steroidal anti-inflammatory drugs (NSAIDs) are widely used by athletes and those who exercise, yet their influence on the molecular responses to exercise remains unclear. Prior studies have often focused on a limited set of molecular pathways, potentially overlooking broader regulator effects of NSAIDs on skeletal muscle signaling. Therefore, we conducted a systems biology study of skeletal muscle biopsies taken before and after exercise, in combination with NSAID consumption, using transcriptomics and metabolomics, to identify differentially enriched pathways and biofunctions.

**Methods:**

We conducted a randomized, counterbalanced, double-masked, crossover trial (NCT05512013) in which 12 healthy adults ingested ibuprofen (IBU, 800 mg), celecoxib (CEL, 200 mg), flurbiprofen (FLU, 100 mg), or placebo (PLA) before a 10 × 10 bout of plyometric exercise. Skeletal muscle biopsies were collected before NSAID consumption and three hours post-exercise. Whole transcriptome profiling was performed using RNA-seq, and the metabolomics profile was assessed via untargeted mass spectrometry. Differential expression analysis and pathway enrichment were used to evaluate NSAID-specific effects across biological domains.

**Results:**

FLU regulated the largest number of differentially expressed transcripts, followed by IBU and CEL. All NSAIDs activated immune-related gene networks and reversed exercise-induced lipid catabolism, with IBU enhancing adaptive immune signaling and CEL modulating both innate and adaptive pathways. Muscle remodeling pathways, including angiogenesis and cell migration, were activated across all NSAIDs, though cachexia-related genes were also upregulated. Interestingly, FLU uniquely upregulated transcripts involved in neuritogenesis.

**Conclusion:**

NSAIDs trigger drug-specific molecular responses in skeletal muscle post-exercise, affecting early recovery through changes in immune, metabolic, and neuronal signaling.

**Supplementary Information:**

The online version contains supplementary material available at https://doi.org/10.1186/s13395-026-00435-4.

## Introduction

NSAIDs are commonly used to manage pain and inflammation [1, 2]. These drugs are categorized by their selectivity for cyclooxygenase (COX) isoforms: COX-1, which is constitutively active, and COX-2, which is inducible in response to exercise or injury [3, 4]. Current evidence suggests that NSAIDs influence the response to exercise in skeletal muscle by inhibiting COX enzymes [5, 6], and these enzymes have been shown to regulate muscle regeneration and muscle protein synthesis (MPS) [5, 7, 8]. However, previous studies have focused on a limited set of molecular pathways, potentially overlooking broader regulator effects of NSAIDs on molecular signaling in skeletal muscle [9–14].

Following acute exercise, transient changes in mRNA expression and metabolites are thought to be the basis of long-term adaptations at the protein level and lead to phenotypical changes in skeletal muscle. With interest in precision medicine on the rise, it is important to understand the differential effects of NSAIDs to develop individualized approaches that improve musculoskeletal health, but these changes must be comprehensively evaluated first [15, 16]. Therefore, the purpose of this study was to determine the transcriptomic and metabolomic response to an acute bout of plyometric exercise with IBU, CEL, or FLU, compared to PLA. We hypothesized that all three NSAIDs would reduce activation of inflammatory, protein synthesis, and mitochondrial pathways compared to PLA.

## Methods

**Fig. 1:**
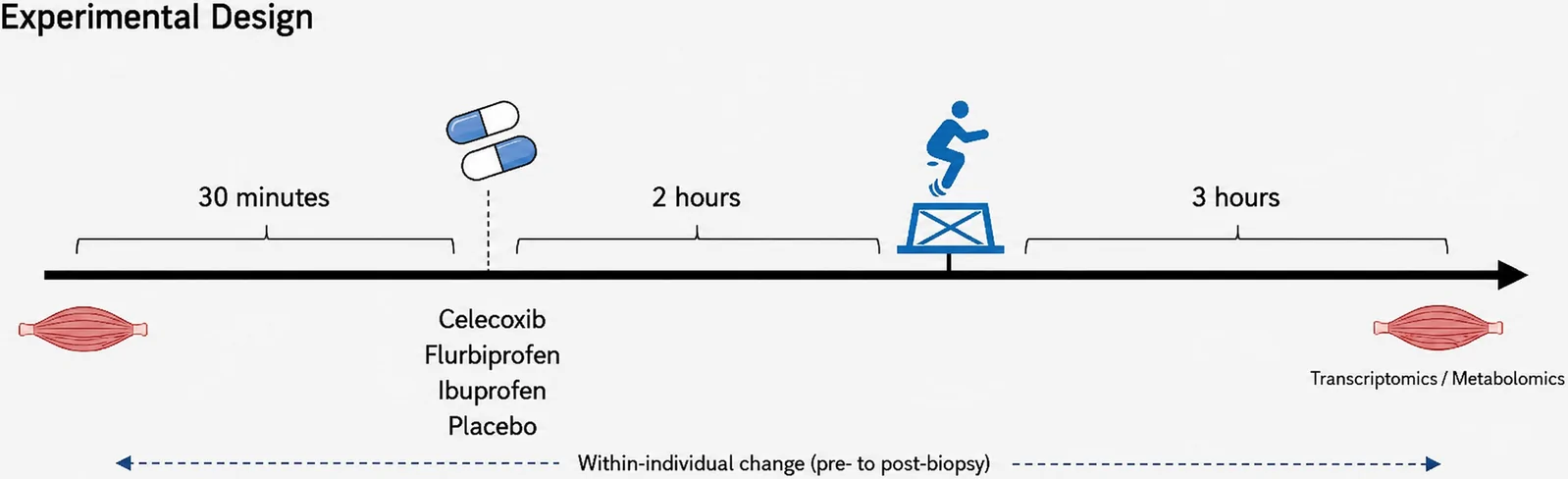
Experimental Design. A muscle biopsy was collected from the vastus lateralis using a Bergstrom biopsy needle before NSAID administration and exercise. Participants then consumed the respective NSAID with a light breakfast, which was matched to each trial. The plyometric exercise bout was conducted two hours after consuming the NSAID or placebo. The exercise consisted of 10 sets of 10 jumps at 40% 1RM with a two-minute rest period between each set. Three hours after the end of exercise, another muscle biopsy was collected in the opposite leg for transcriptomics and metabolomics analysis. Participants consumed each drug in a counterbalanced, double-masked, randomized-crossover order

This study was preregistered on clinicaltrials.gov (NCT05512013). We used a randomized, double-masked, counter-balanced, crossover design to assess the impact of a single dose of IBU (800 mg), CEL (200 mg, CEL), FLU (100 mg), or a PLA as previously described [17, 18]. After familiarization and baseline 1RM measurements, participants proceeded with the four experimental trials (Fig. 1). Each trial was separated by at least a one week washout to allow recovery from the prior plyometric exercise bout and to minimize pharmacologic effects before the next condition. 12 healthy adults (10 males, two females) were included in the study. Inclusion criteria were ages 18–42 years old, exercising at least twice per week, and willing to discontinue the use of supplements. Exclusion criteria included medical conditions affecting kidneys, endocrine, or cardiovascular systems; bone metabolism disorders; NSAID allergies; past gastric ulcers or NSAID-induced gastric distress. Informed consent was obtained from participants, and the study was approved by the US Army Medical Research Development Command Institutional Review Board, adhering to DOD instruction 3216.02 and 32 CFR Part 219 guidelines on human subject protection.

Pre-exercise, a muscle biopsy was collected from the vastus lateralis using a Bergstrom biopsy needle and immediately frozen in liquid nitrogen, then stored at -80˚C until further analysis. Participants then consumed the respective NSAID with a light breakfast standardized within participant across all four experimental trials that was provided as 8 kcal/kg body weight with 60–65% carbohydrate, 10–15% protein, and 20–25% fat. The plyometric exercise bout was conducted two hours after consuming the NSAID or PLA. For each of the four experimental trials, participants performed a single bout of plyometric exercise at 40% of their 1RM on the Plyo-press. The exercise consisted of 10 sets of 10 jumps at a with a two-minute rest period between each set [19]. Three hours after the end of exercise, another muscle biopsy was collected in the opposite leg. Two aliquots of muscle biopsies were used for RNA-seq assays and liquid chromatography/ mass spectroscopy (LC/MS) metabolomics assays, respectively.

### RNA-seq transcriptomics

The mRNA Libraries were prepared using the Illumina Stranded Total RNA Prep, Ligation with Ribo-Zero Plus kit (Illumina, Inc., San Diego, CA, USA), following the manufacturer’s protocol with 100 ng of total RNA. The resulting mRNA libraries were validated through the 4200 TapeStation System (Agilent Technologies, Santa Clara, CA, USA), cleaned up using SPRIselect beads (Beckman Coulter, Inc., Bream, CA, USA), and quantified using the Qubit 3.0 Fluorometer. The libraries were normalized to ~4nM, pooled, diluted, and sequenced on the HiSeq 4000 (Illumina, Inc.) platform using the HiSeq 3000/4000 150 cycle pair-end reagents. Each read was trimmed by trimmomatic v0.39 [20] with the following parameters: ILLUMINACLIP: TruSeq3-PE-2.fa:2:30:10 SLIDINGWINDOW:4:20 MINLEN:36, then mapped to the top level ensemble human genome using STAR v2.7.11b [21]. The GeneCount files was generated and subsequently processed using prcomp function from the stats package in R library to create the Principal Component Analysis (PCA) plots.

For the differential expression analysis, the samples collected from each of these four groups were sorted into two sub-groups: (a) pre-exercise and (b) post-exercise treatment. DESeq2 package was used to calculate log2 fold change (lfc) following this equation-.

$\:lfc={\left(\frac{Post-exercise}{Pre-exercise}\right)}_{n}$, where *n* represents one of four experimental groups [22]. The differentially expressed genes (DEGs) were identified as those with an FDR corrected *p*-values < 0.05. The DEGs were visualized using heatmaps created by the ComplexHeatmaps in R package [23]. The RNA-seq data has been archived in NCBI SRA database with ID: SUB15537512.

### LC/MS based metabolomics

The second aliquot of the homogenized muscle biopsy sample was used for an untargeted metabolomics assay using a Quadruple Time-of-Flight mass spectrometer (Q-TOF MS) (Waters, Inc., Milford, MA) following a previously documented protocol [24, 25]. Briefly, 50 mg of the homogenized tissue was dissolved in chilled extraction buffer (50% methanol in water), and the protein-containing phase was removed as the proteins were precipitated by the addition of equal volumes of acetonitrile. Five microliters of the protein-free sample was injected onto a reverse-phase 50 × 2.1 mm Acquity 1.7-µm C18 column (Waters Corp) using an Acquity Ultra Performance Liquid Chromatography (UPLC) system (Waters) with a gradient mobile phase consisting of 2% acetonitrile in water mixed with 0.1% formic acid (Solvent A) and 2% water in acetonitrile mixed with 0.1% formic acid (Solvent B); and the column was resolved for 10 min at a flow rate of 0.5 mL/min. The gradient consisted of 100% A for 0.5 min with a ramp up curve 6–100% B from 0.5 to 10 min.

The column eluent was introduced directly into the G2-S mass spectrometer by electrospray. Mass spectrometry was performed on a quadrupole-time-of-flight mass spectrometer operating in either negative or positive electrospray ionization. Positive mode had a capillary voltage of 3.00 kV and a sampling cone voltage of 30 V. Negative mode had a capillary voltage of 2.75 kV and had a sampling cone voltage of 20 V. The desolvation gas flow was set to 600 L/hour, and the desolvation temperature was set to 500 °C. The cone gas flow was 25 L/h and the source temperature was 100 °C. The data was acquired in the sensitivity MS mode with a scan time of 0.300 s and an interscan time of 0.014 s. Accurate mass was maintained by infusing Leucine Enkephalin (556.2771 m/z) in 50% aqueous acetonitrile (1.0 ng/mL) at a rate of 10 µL/min via the Lockspray interface every 10 s. Data was acquired in Centroid mode with a 50.0 to 1200.0 m/z mass range for TOF MS scanning.

The untargeted data acquired were first converted to the NetCDF unified data format using the Databridge tool in MassLynx (Waters Corporation, Milford, MA). An in-house implementation of the XCMS R package (Scripps Institute, La Jolla, CA) was used for peak detection with an ordered bijective interpolated warping algorithm utilized for retention time correction, and parameters optimized using the Isotopologue Parameter Optimization (IPO) R package. The mass to charge ratio and retention time (mzrt) features were normalized based on the internal standards (debrisoquine and 4-nitrobenzoic acid present in the extraction solution in positive and negative modes, respectively) as well as QC-RLSC (QC robust LOESS signal correction) normalization.

The normalized spectral features were processed by R-scripts to first determine differentially expressed peaks, as we used the same method as described earlier to find DEGs. Within each of the four experimental groups, two sub-groups were created by sorting the samples extracted at pre-exercise and post-exercise timepoints, respectively. Subsequently, differentially expressed spectral features were identified using the threshold *p* < 0.05. These curated peaks further were annotated using CEU Mass Mediator database and the molecules were screened based on the following guideline [26]: (a) ppm error < 10; (b) chemical formula comprised of the adducts: +H, -H, +Na, +K, +NH4, -Cl were included; (c) spectral featured annotated by Human Metabolome Database (HMD) were included; and (d) chemical type of one of the following categories: (i) endogenous mammalian; (ii) drugs; (iii) toxicant; and (iv) reagents were included. These were identified as differentially expressed metabolites (DEMs).

### Knowledge integration

Gene and metabolite knowledge integration was carried out at the network level. The DEGs and DEMs were uploaded to the IPA platform (QIAGEN Inc., Valencia, CA) to curate the significantly enriched biological networks. The networks were down-selected based on the criteria set at the hypergeometric threshold of *p* < 0.05, z-score > |1.5|, and the total number of differentially expressed molecules enriching the network > 5. Here, the differentially expressed molecules were defined as the combination of the DEGs and DEMs. The networks showing z-score > 1.5 and < -1.5 were defined as activated and inhibited networks, respectively.

## Results

Participants included two females and ten males with an average age of 25 ± 6y, average height of 174.6 ± 10.2 cm, and average weight of 82.4 ± 14.8 kg. RNA quality was assessed using the Agilent TapeStation 4200 system to determine the RNA Integrity Number (RIN). Overall, the RIN mean and median was 8.5 and 8.6, respectively, with standard deviation of ± 0.56.

### PCA plot highlights differential perturbations caused by NSAIDs

Genomic data was imported into R for analysis. Genes were filtered for: a variance greater than 0, 30% of all samples had a count present, total gene count greater or equal to 1.5x the sample size (99), a percent CV greater than 20%, and ensemble IDs that have a gene symbol.

PCA of the transcriptomic data showed treatment-associated differences across the four conditions (Figure S1A). In this analysis, PC1 and PC2 explained 12.53% and 7.52% of the total variance in the mRNA dataset, respectively. A similar pattern was observed in the metabolomics PCA (Figure S1B), where PC1 and PC2 explained 25.26% and 11.60% of the total variance in the metabolite dataset, respectively.

Differential expression analysis of the transcriptomic data identified 598 DEGs in the IBU condition, including 385 upregulated and 213 downregulated genes; 523 DEGs in the CEL condition, including 463 upregulated and 60 downregulated genes; 458 DEGs in the FLU condition, including 215 upregulated and 243 downregulated genes; and 244 DEGs in the PLA condition, including 186 upregulated and 58 downregulated genes. Similarly, differential metabolite analysis identified DEMs in the IBU condition, including 299 increased and 284 decreased metabolites; 765 in the CEL condition, including 473 increased and 292 decreased metabolites; 482 in the FLU condition, including 182 increased and 300 decreased metabolites; and 932 in the PLA condition, including 68 increased and 864 decreased metabolites.

### Differential impacts of NSAIDs on gene-metabolite biofunctions

**Fig. 2:**
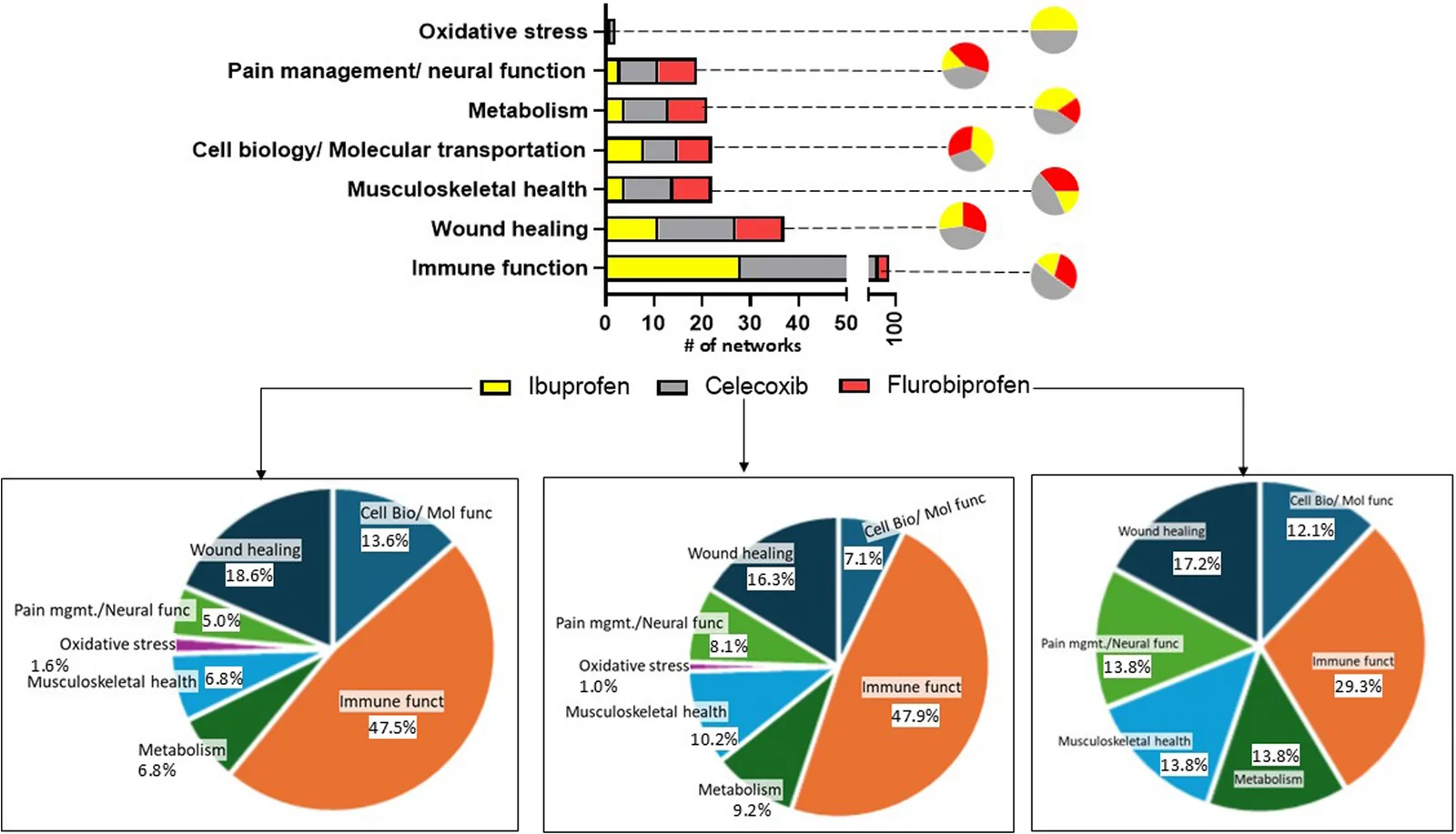
Impacts of NSAIDs on the biofunction superfamilies. The bar chart represents the number of networks that are impacted by each of the drugs. Each bar is linked to a pie chart showing the degree of impact of each drug on individual bionetwork superfamily. At the bottom of the bar graph, three pie charts show the comparative distributions of the total number of bionetwork superfamily impacted by these three NSAIDs

Bar and pie charts summarized the differential regulations of the biofunctions regulated by the NSAIDs (Fig. 2). Following plyometric exercise, immune functions related networks emerged as the largest gene-metabolite network superfamily regulated by NSAIDs, and 51% of these differentially regulated immune functions were regulated by CEL alone. Wound healing-related gene-metabolite networks emerged as the second most perturbed network superfamily, and three NSAIDs equally regulated this network superfamily. Pie charts of individual NSAIDs revealed that ~ 48% of the networks perturbed by CEL were linked to immune regulation. Likewise, 48% of those networks, which were perturbed by IBU were linked to immune regulation. The pie chart linked to FLU was almost proportionally sorted among six network superfamilies, marginally led by immune regulation (29%) and wound healing (17%).

### Immune regulation

**Fig. 3:**
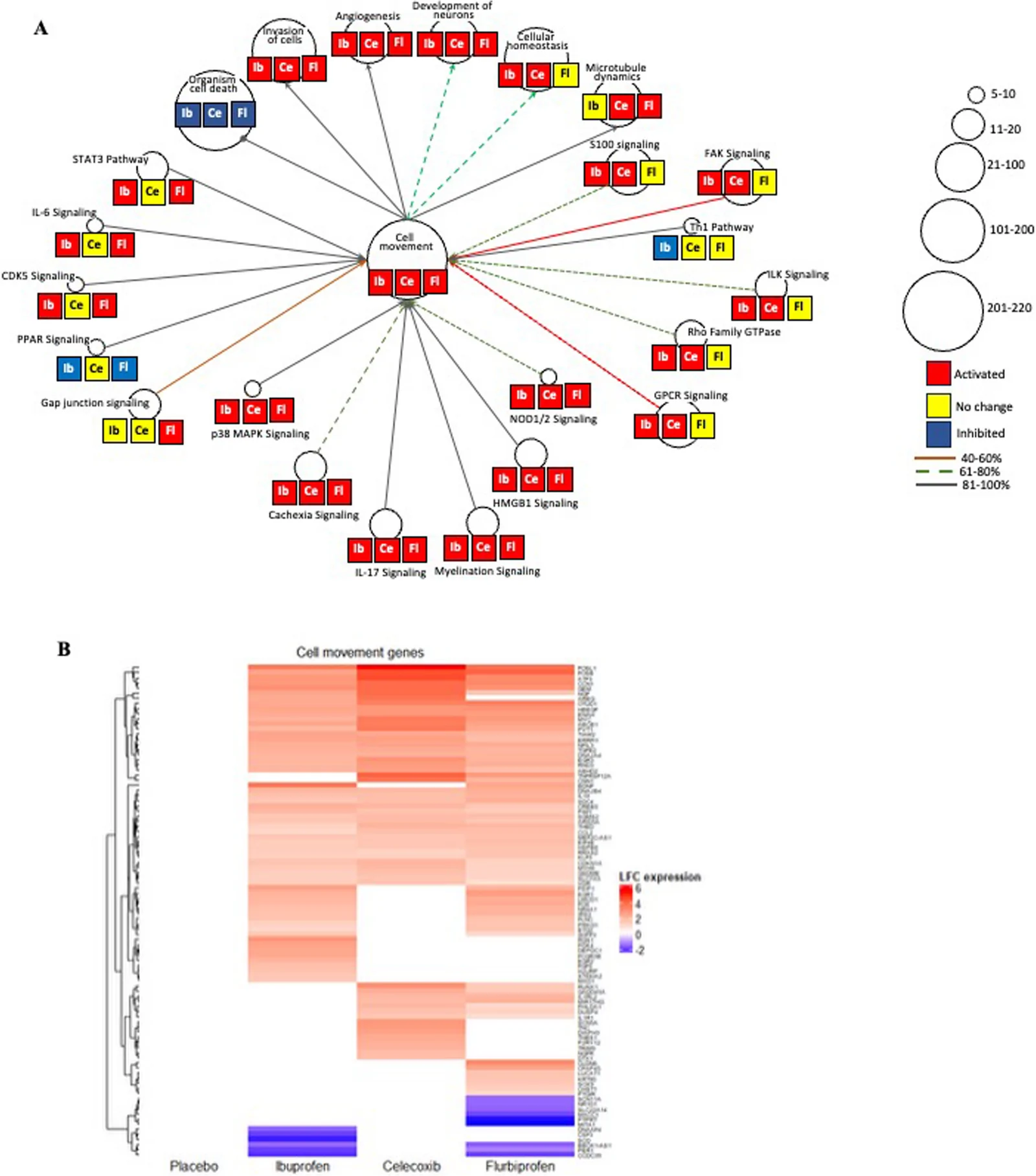
NSAIDs regulation of cell movement network, the most enriched biofunction in this study. **A** The regulation profile illustrates a group of co-regulated networks associated with the cell movement network, which is positioned at the center of this cluster. Node sizes correspond to the number of DEGs and DEPs enriching each respective network. Arrow-headed edges indicate activated relationships between nodes, while blunt-headed edges denote inhibited interactions. Each node contains three boxes representing regulation levels induced by the three drugs: ibuprofen (Ib), celecoxib (Ce), and flurbiprofen (Fl). Regulation profiles are color-coded as follows: up-regulated (red), down-regulated (blue), and no change (yellow). **B** Hierarchical clustering of DEGs and DEPs that enriched the cell movement network

More than half of all regulated pathways under IBU or CEL were associated with immune functions (Table S1). IBU predominantly activated adaptive immune transcripts, demonstrated by upregulation of genes involved in T-cell development and activation. In contrast, CEL induced a broader immune response encompassing both adaptive and innate immunity, including enhanced signals for lymphocyte proliferation, lymphocyte migration, and leukocyte. FLU affected immune pathways in a more balanced manner, contributing to a wide range of network changes across multiple domains. Despite these drug-specific differences, common immune regulatory patterns emerged. All three NSAIDs co-activated a gene network related to cell movement (migration), which is enriched by the largest number of DEGs among the networks (Fig. 3). This network was activated by all three NSAIDs and co-perturbed by 23 sub-networks. Network linked to organismal cell death shared the largest number of downregulated DEGs with the cell movement network. On the other hand, the networks linked to angiogenesis and the development of neurons, which were downstream regulators of the cell movement network and all were unregulated by NSAIDs.

### Muscle health gene-metabolite networks differentially perturbed by NSAIDs

**Fig. 4:**
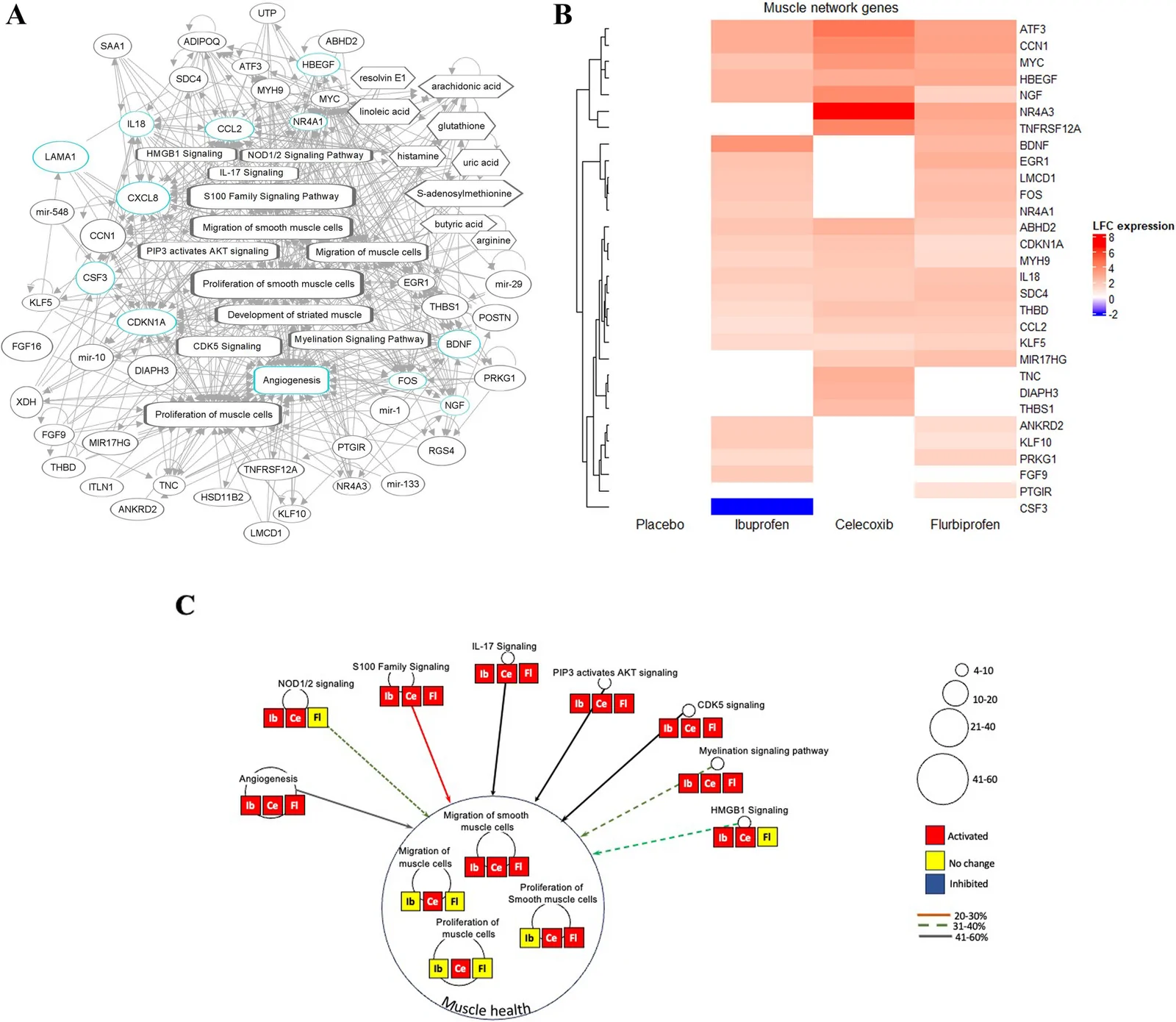
Muscle health gene-metabolite network differentially perturbed by NSAIDs. **A** Muscle health network is enriched by 55 nodes representing genes (ovals) and metabolites (hexagons), and 24 co-perturbed networks (rectangles). **B** Hierarchical clustering of genes linked to muscle health network. **C** Regulation profile of a cluster of co-regulated networks linked to muscle health. The core of the cluster contains four networks linked to migration of muscle cells, migration of smooth muscle cells, proliferation of muscle cells, and proliferation of smooth muscle cells, respectively. The size of the nodes encompassing the network and the color of the edges connecting the nodes represent the number of genes enriching the function and the percentage of overlapping genes, respectively. Arrow-headed edges indicate activated relationships between nodes, while blunt-headed edges denote inhibited interactions. Each node contains three boxes representing regulation levels induced by the three drugs: ibuprofen (Ib), celecoxib (Ce), and flurbiprofen (Fl). Regulation profiles are color-coded as follows: up-regulated (red), down-regulated (blue), and no change (yellow)

All NSAIDs induced significant changes in gene-metabolite networks associated with muscle structural remodeling and repair (Fig. 4). Acute plyometric exercise inhibited the apoptosis of muscle cells and cell cycle progression of muscle cells. Post-NSAID treatment, an integrated gene–metabolite cluster (comprising ~ 55 molecules) linked to muscle cell migration and proliferation was strongly perturbed. NSAID treatment also consistently activated pathways associated with muscle catabolism. Notably, a cachexia network (muscle wasting) was activated by IBU, CEL, and FLU. Several transcripts linked to muscle atrophy and protein breakdown showed increased expression in all NSAID groups, indicating a catabolic stimulus in the presence of these drugs. This occurred even as pathways promoting cellular homeostasis and survival were active, highlighting the mixed nature of the muscle’s response.

### Neuronal signaling

**Fig. 5:**
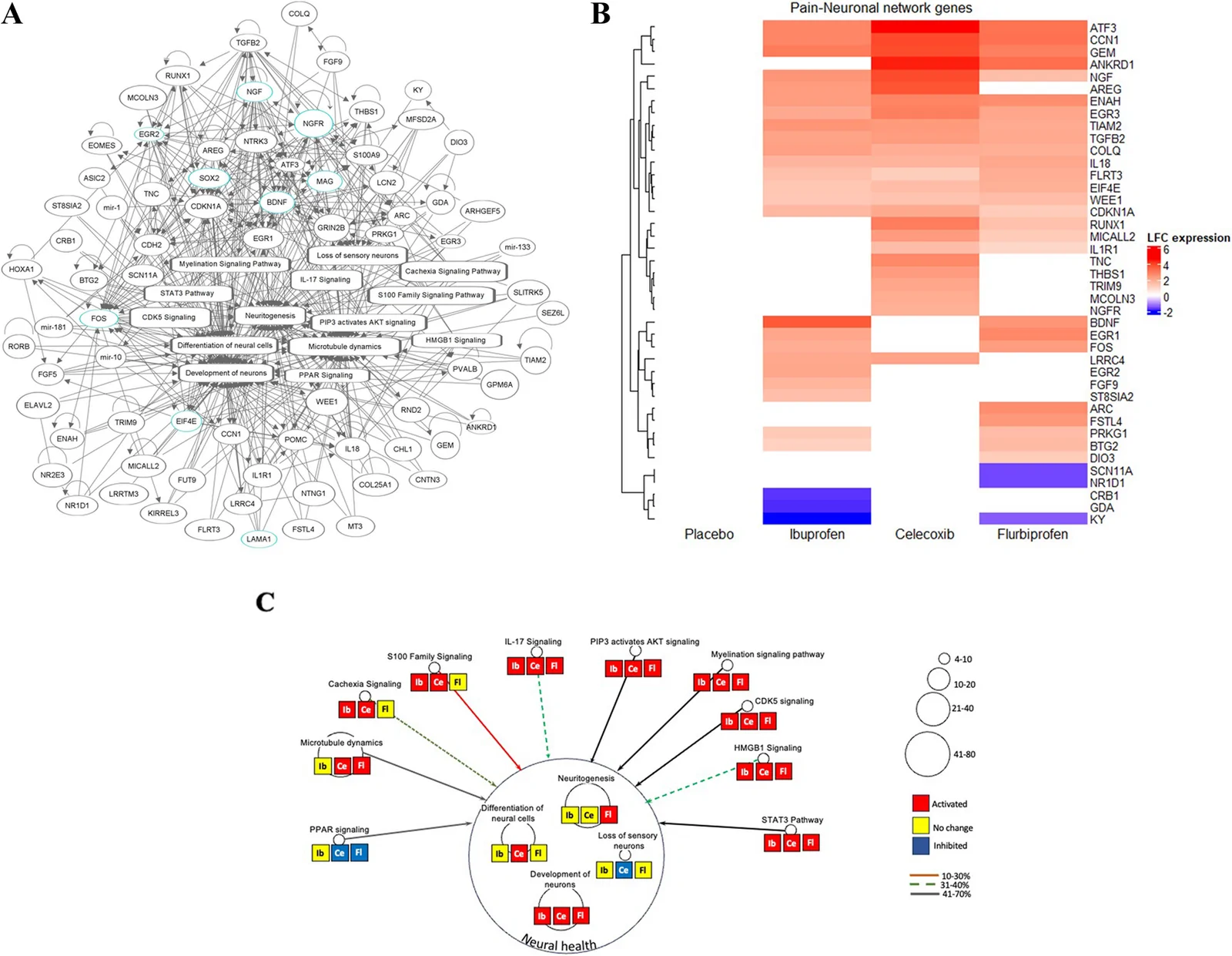
Neural health gene-metabolite network differentially perturbed by NSAIDs. **A** Neural health network is enriched by 72 nodes representing genes (ovals) and metabolites (hexagons), and 14 co-perturbed networks (rectangles). **B** Hierarchical clustering of genes linked to neural health network. **C** Regulation profile of a cluster of co-regulated networks linked to muscle health. The core of the cluster contains four networks linked to neuritogenesis, differentiation of neuronal cell, development of neurons and loss of sensory neurons, respectively. The size of the nodes encompassing the network and the color of the edges connecting the nodes represent the number of genes enriching the function and percentage of overlapping genes, respectively. Arrow-headed edges indicate activated relationships between nodes, while blunt-headed edges denote inhibited interactions. Each node contains three boxes representing regulation levels induced by the three drugs: ibuprofen (Ib), celecoxib (Ce), and flurbiprofen (Fl). Regulation profiles are color-coded as follows: up-regulated (red), down-regulated (blue), and no change (yellow)

Gene-metabolite networks related to neuronal development and signaling were influenced by exercise and differentially influenced by NSAIDs. All three NSAIDs activated neurogenesis-related gene networks (Fig. 5).

### Lipid metabolism

**Fig. 6:**
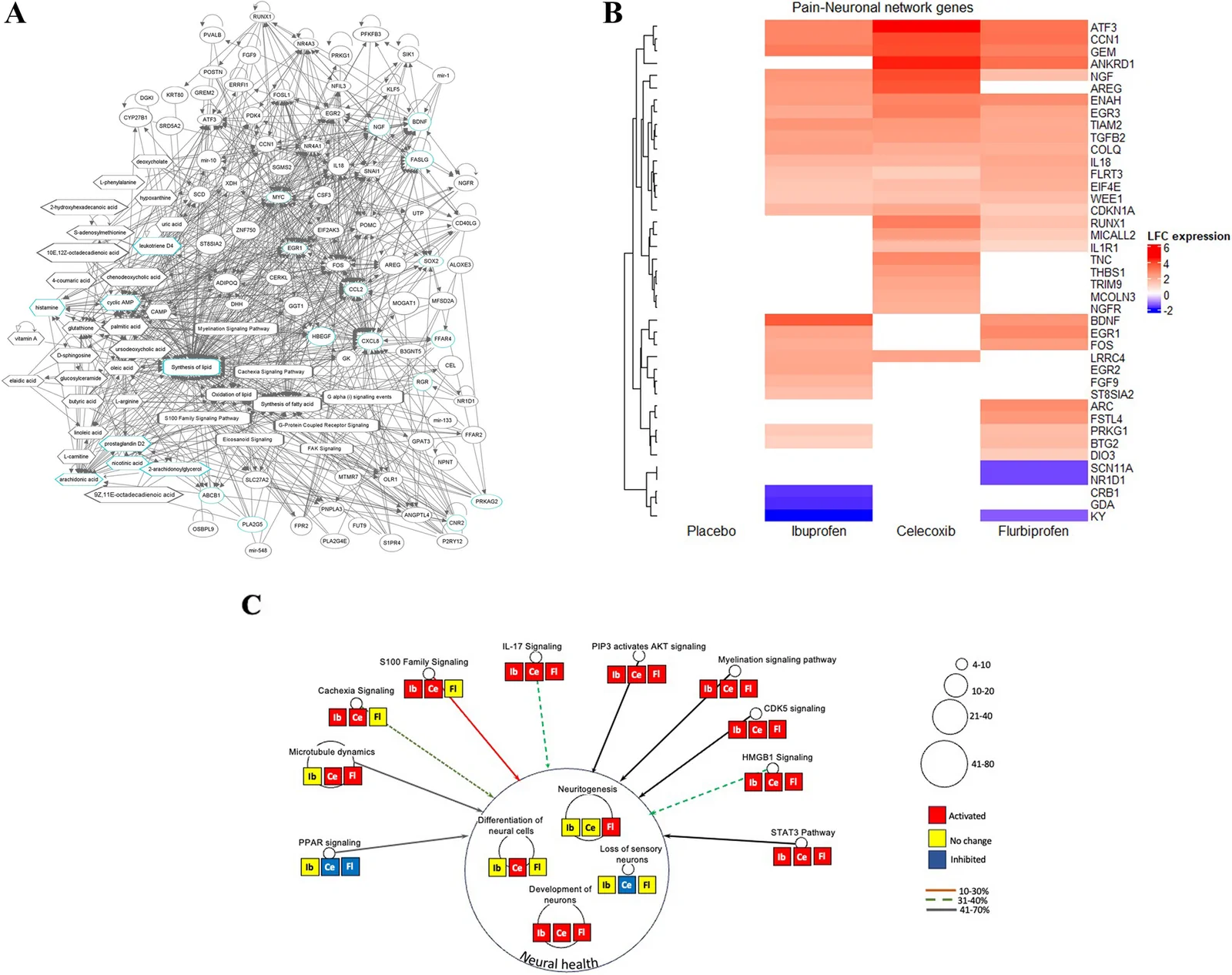
Lipid metabolism gene-metabolite network differentially perturbed by NSAIDs. **A** Lipid metabolism network is enriched by 110 nodes representing genes (ovals) and metabolites (hexagons), and 9 co-perturbed networks (rectangles). **B** Hierarchical clustering of genes linked to lipid metabolism network. **C** Regulation profile of a cluster of co-regulated networks linked to lipid metabolism. The core of the cluster contains two networks linked to synthesis of fatty acids and synthesis of lipids, respectively. The size of the nodes encompassing the network and the color of the edges connecting the nodes represent the number of genes enriching the function and percentage of overlapping genes, respectively. Arrow-headed edges indicate activated relationships between nodes, while blunt-headed edges denote inhibited interactions. Each node contains three boxes representing regulation levels induced by the three drugs: ibuprofen (Ib), celecoxib (Ce), and flurbiprofen (Fl). Regulation profiles are color-coded as follows: up-regulated (red), down-regulated (blue), and no change (yellow)

Acute plyometric exercise produced a prominent metabolic signature, with lipid metabolism emerging as one of the major biological domains altered by NSAID treatment. In the placebo condition, the post-exercise response was characterized with reduced lipid synthesis and increased lipid breakdown. In contrast, NSAID conditions shifted this response toward lipid synthesis and lipid storage-related signaling. This effect was most evident for CEL, which activated networks related to synthesis of fatty acids and synthesis of lipids, whereas ibuprofen and flurbiprofen produced more selective lipid-network changes. These differences suggest that NSAIDs do not uniformly affect post-exercise lipid metabolism but produce drug-specific metabolic signatures that may reflect differences in COX selectivity or downstream regulation of substrate handling (Fig. 6).

### Angiogenesis

**Fig. 7:**
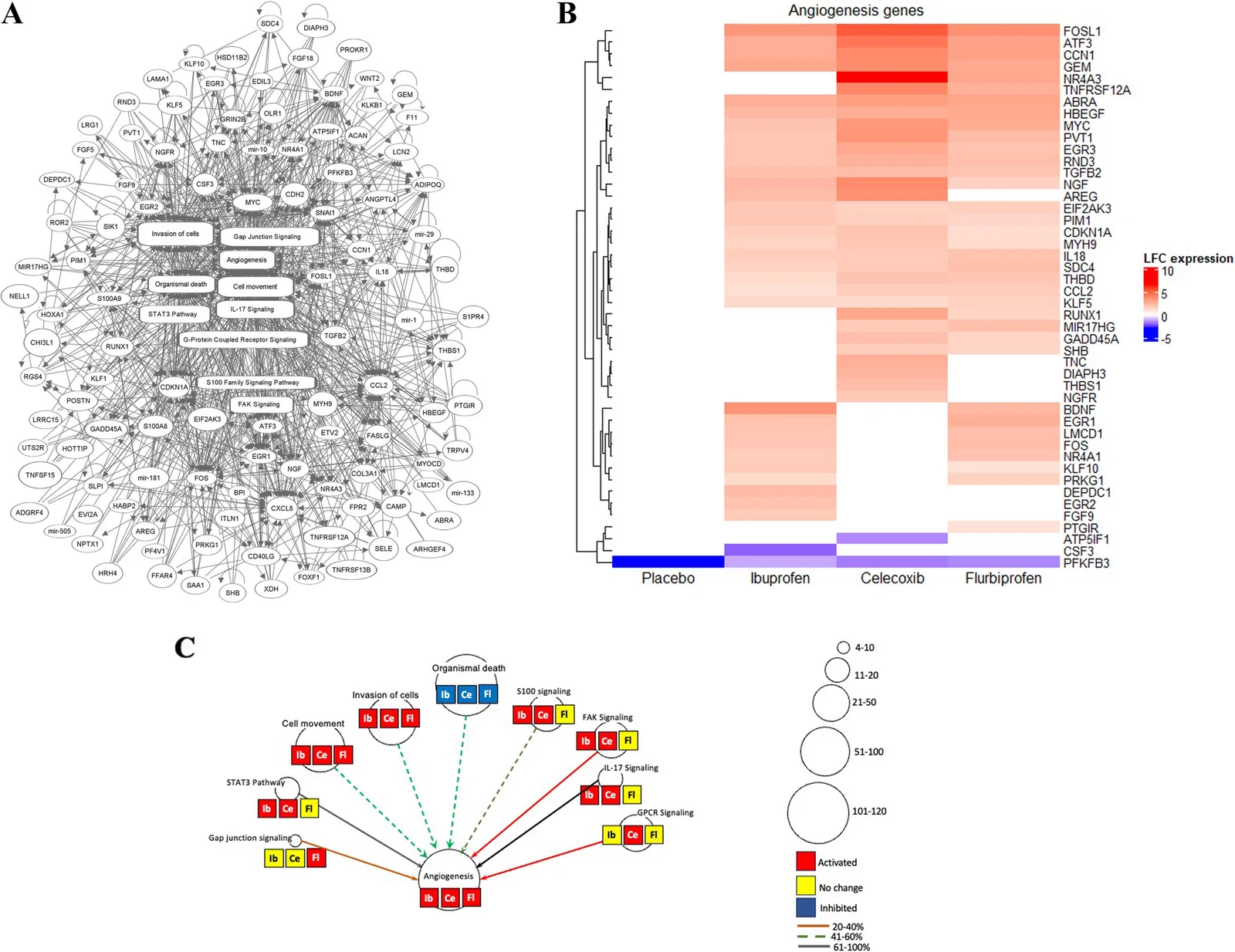
Angiogenesis gene-metabolite network differentially perturbed by NSAIDs. **A** Lipid metabolism network is enriched by 100 nodes representing genes (ovals) and metabolites (hexagons), and 10 co-perturbed networks (rectangles). **B** Hierarchical clustering of genes linked to angiogenesis network. The legend is at the right-hand side. **C** Regulation profile of a cluster of co-regulated networks linked to angiogenesis. The size of the nodes encompassing the network and the color of the edges connecting the nodes represent the number of genes enriching the function and percentage of overlapping genes, respectively. Arrow-headed edges indicate activated relationships between nodes, while blunt-headed edges denote inhibited interactions. Each node contains three boxes representing regulation levels induced by the three drugs: ibuprofen (Ib), celecoxib (Ce), and flurbiprofen (Fl). Regulation profiles are color-coded as follows: up-regulated (red), down-regulated (blue), and no change (yellow)

NSAID administration induced regulation of angiogenesis-related gene networks following plyometric exercise (Fig. 7). All three NSAIDs activated the core angiogenesis network, whereas the PLA condition showed a relative lack of activation. IBU and CEL also enhanced co-regulated networks involved in cell movement, invasion of cells, and S100 signaling, which are integral to endothelial activation and vascular sprouting. CEL elicited the most widespread transcriptional response within these pathways. Ibuprofen similarly promoted expression changes associated with microvascular adaptation, including enrichment of focal adhesion kinase (FAK) and STAT3 signaling. In contrast, FLU produced a more modest angiogenic response, but selectively activated genes associated with gap junction signaling, which may support intercellular coordination during capillary formation.

## Discussion

This study provides a multi-omics, systems biology analysis of the skeletal muscle response to acute plyometric exercise with three different NSAIDs compared to a placebo in active healthy adults. Using transcriptomic and metabolomic profiling, we found that IBU, CEL, and FLU each regulated overlapping and distinct biological networks in human skeletal muscle, spanning immune regulation, tissue remodeling, metabolism, and neuroplasticity. These findings align with, but more importantly extend on prior studies, offering integrative insights into how NSAIDs shape early-phase muscle recovery following exercise.

Consistent with previous studies, our data confirms that NSAIDs exert prominent effects on immune-related gene networks and expands the literature to include novel pathways [6, 8]. Pro-inflammatory cytokine networks, notably those centered on IL-17, were activated across all NSAID conditions. However, we found that NSAIDs do not suppress inflammation but rather shift the immune signature toward a mixed phenotype involving both pro-inflammatory and anti-inflammatory signaling, evidenced by the co-activation of S100 and ILK signaling by IBU and CEL. CEL upregulated a muscle health gene cluster enriched for NOD and HMGB1, factors previously associated with regenerative responses in dystrophic muscle [27]. NOD1 and NOD2 activate NF-κB and MAPK which cause pro-inflammatory cytokine production and influence macrophage and satellite cell activity during muscle regeneration [28]. HMGB1, a nuclear protein released in response to cellular stress or necrosis, amplifies inflammatory signaling through interactions with TLR4, RAGE, and NOD-like receptors, further promoting leukocyte recruitment and repair cascades [29]. Interestingly, CEL did not activate the IL-6 or STAT3 signaling pathways, which expands previous data suggesting COX-2 inhibitors can blunt some, but not all, inflammation pathways in skeletal muscle [7]. These patterns demonstrate key differences and similarities between NSAIDs that play a role in cell migration and inflammation.

A novel aspect of our findings is that NSAIDs influenced neuronal development-related gene networks. FLU uniquely activated neuritogenesis, which is similar to neuromuscular reinnervation models [30]. This suggests promotion of the neuron repair or growth (e.g., reinnervation of muscle fibers). CEL and FLU both activated microtubule signaling, likely supporting cytoskeletal remodeling in motor neurons. These pathways are involved in neuronal remodeling and neuromuscular junction plasticity, raising the possibility that NSAIDs could affect neural components of recovery, particularly under conditions involving denervation, motor unit remodeling, or fatigue [31]. A general neuron development network was activated under all NSAID conditions, implying a shared tendency to facilitate neural adaptive processes in muscle after plyometric exercise. Pathways related to the loss of sensory neurons (included in the neuronal network analysis) were not changed by IBU or FLU, but CEL inhibited this network. Together, our data indicate a shift towards neuroprotective or pro-neurogenic responses with NSAID use, with FLU showing the strongest promotion of neural growth-related networks. However, these interpretations are mechanistic and hypothesis-generating; further studies are needed to link these signals with functional neuromuscular outcomes since this is the first time, to our knowledge, that NSAIDs have been reported to influence neuronal signaling.

All three NSAIDs appeared to counteract the typical lipid catabolic response to exercise by activating the synthesis of lipids. Interestingly, CEL activated networks that control the synthesis of fatty acids, but IBU and FLU did not change them. This aligns with prior studies showing that NSAID use can alter substrate metabolism during exercise recovery. For example, Markworth et al. (2013) demonstrated that IBU intake modulated lipid mediator profiles involved in inflammatory resolution and metabolic signaling [32] while Cardinale et al. (2017) found that IBU reduced mitochondrial function but not mitochondrial content in healthy young individuals [33]. Moreover, NSAIDs may disrupt PPAR and prostaglandin pathways that regulate lipid oxidation and substrate flexibility [6, 34]. Furthermore, NSAID-induced disruption of lipid signaling may impair metabolic flexibility, promoting carbohydrate over the use of fat. This altered metabolic profile may have implications for recovery and adaptation, potentially compromising the efficiency of fuel utilization and long-term muscle adaptations.

All three NSAIDs activated angiogenesis pathways, which is a hallmark of muscle remodeling [35]. This supports and refines observations from others who have found that chronic NSAID intake can blunt hypertrophic responses in young adults while preserving or even enhancing early muscle repair in older adults [5, 36]. Network topology and heatmap analyses revealed that NSAID-induced angiogenic programming involved increased expression of canonical regulators such as *FOSL1*, *CCN1*, and *TGFB2*, along with downstream effectors implicated in endothelial migration and cytoskeletal remodeling, including *CDKN1A* and *MYH9* [37–39]. Notably, *NR4A3* was upregulated with CEL and FLU but not IBU and plays a role in exercise-induced vascular remodeling [40]. Although several angiogenic genes were commonly regulated, the magnitude and network structure of the response varied by drug, suggesting NSAID-specific changes in vascular adaptation. Collectively, these results indicate that NSAIDs alter angiogenesis-related signaling following exercise, with IBU and CEL promoting extensive vascular gene responses compared to PLA.

While our data provides novel mechanistic insight into acute NSAID effects in human skeletal muscle, it is important to recognize the model-specific context. Earlier studies used animal models of injury, aging, or chronic NSAID administration, which complicate direct comparison [12, 13]. A major strength is the randomized, double-masked, counterbalanced crossover design, which allowed each participant to complete all four treatment conditions and serve as their own control. Experimental trials were separated by at least one week, and pre-exercise meals, testing procedures, exercise timing, and biopsy collection were standardized within participants to reduce biological and technical variability. The use of paired skeletal muscle biopsies, RNA-seq, untargeted metabolomics, and integrated pathway analysis also provided a broad view of the early molecular response to NSAID use and exercise. A key limitation of our study is the absence of a non‑exercise control condition, which prevents us from fully distinguishing NSAID‑specific effects from exercise‑independent changes; however, this is mitigated by our focus on post‑exercise molecular responses, as the primary goal was to characterize how NSAIDs modulate exercise‑induced signaling and metabolism rather than their effects at rest. There are also several analytical limitations that should be considered. Although the parent trial used a crossover design, the omics analyses were performed within each treatment condition by contrasting post-exercise samples against pre-exercise samples. This approach allowed us to characterize treatment-specific molecular perturbations, but it limits formal inference regarding direct between-NSAID comparisons and treatment-by-time interactions. In addition, the statistical thresholds differed across omics platforms. RNA-seq differential expression analyses used FDR-corrected p values, whereas the untargeted metabolomics analysis used a nominal *p* < 0.05 threshold before metabolite annotation and downstream network integration. Therefore, the integrated gene-metabolite networks combine transcriptomic features identified using a more conservative FDR-corrected approach with metabolomic features identified using a less conservative exploratory threshold. Finally, the cohort was small and predominantly male, limiting the ability to evaluate sex-specific molecular responses.

Nonetheless, our findings echo key principles: (1) NSAIDs rarely act as simple suppressors of muscle adaptation, and (2) their molecular effects are context dependent. For example, Trappe et al. showed that NSAIDs enhanced resistance training gains in older adults but had little effect in younger cohorts [41, 42]. Our results suggest that such differential outcomes may reflect changes in the immune-muscle axis with aging. On the other hand, Cornu et al. [43] noted in their meta-analysis that NSAIDs may influence performance under certain, but not all, conditions, suggesting that the impact of NSAIDs is likely dependent on factors such as exercise type, intensity, and individual responsiveness, including underlying inflammation or recovery demands.

In conclusion, NSAIDs induce broad transcriptional and metabolic reprogramming in human skeletal muscle following exercise, with shared and unique effects across drug types. These changes span immune, vascular, neuronal, and metabolic systems, suggesting NSAID use can both support and impede recovery processes. Based on emerging molecular evidence, NSAID use around exercise could be individualized depending on recovery goals and health context. While short-term NSAID use may not impair muscle adaptation in healthy adults, chronic or prophylactic use could blunt beneficial remodeling or alter metabolic responses. For example, CEL may be preferable in individuals needing enhanced vascular or tissue repair support due to its COX-2 selectivity and angiogenic signaling effects, whereas FLU could be considered in cases involving neuromuscular pain or reinnervation concerns given its effects on neuritogenesis. In contrast, IBU may offer broad symptomatic relief but appears more likely to disrupt redox-sensitive pathways and lipid metabolism. Future studies should address chronic NSAID use and potential sex differences.

## Supplementary Information

Supplementary Material 1: Principal component analysis (PCA). Each node represents an individual sample, with groups delineated by 95% confidence ellipses. The bar and whisker plots along PC1 and PC2 illustrate the variance within treatment groups- samples collected pre- and post-exercise across the four treatment conditions: ibuprofen, celecoxib, flurbiprofen, and placebo. (A) whole gene transcriptome profiles and (B) untargeted metabolomics profile in human skeletal muscle.

Supplementary Material 2.

## Data Availability

The data from the current study are only available with an approved data sharing agreement requested through the corresponding author due to institute regulations. The RNA-seq data has been archived in NCBI SRA database with ID: SUB15537512.
